# Knock-Down of Core Proteins Regulating MicroRNA Biogenesis Has No Effect on Sensitivity of Lung Cancer Cells to Ionizing Radiation

**DOI:** 10.1371/journal.pone.0033134

**Published:** 2012-03-30

**Authors:** Olga Surova, Nadeem Shahzad Akbar, Boris Zhivotovsky

**Affiliations:** 1 Institute of Environmental Medicine, Division of Toxicology, Karolinska Institutet, Stockholm, Sweden; 2 Faculty of Fundamental Medicine, MV Lomonosov Moscow State University, Moscow, Russia; National Cancer Institute, United States of America

## Abstract

Recent studies underline the important role of microRNAs (miRNA) in the development of lung cancer. The main regulators of miRNA biogenesis are the ribonucleases Drosha, Dicer and Ago2. Here the role of core proteins of miRNA biogenesis machinery in the response of human non-small and small cell lung carcinoma cell lines to treatment with ionizing radiation was assessed. We found that Drosha and Dicer were expressed at higher levels in radioresistant but not in sensitive cell lines. However, down-regulation of either Dicer or Drosha had no effect on the sensitivity of cells to irradiation. Elimination of components of the RNA-induced silencing complex Ago2 and Tudor staphylococcal nuclease also did not sensitize cells to the same treatment. Thus, modulation of miRNA biogenesis machinery is not sufficient to increase the radiosensitivity of lung tumors and other strategies are required to combat lung cancer.

## Introduction

Lung cancer (LC) is a leading cause of cancer mortality worldwide in both women and men. There are two main types of this neoplasia, small cell lung carcinoma (SCLC) and non-small cell lung carcinoma (NSCLC), which differ considerably in their histopathological features and responses to therapy. Ionizing radiation, alone or in combination with surgery or chemotherapy, is an effective treatment for many cancers, including LC. However, both intrinsic and acquired tumor radioresistance greatly reduce the efficacy of radiotherapy for NSCLC and SCLC and often lead to relapse and metastasis. Therefore, it is of great importance to explore the molecular mechanisms underlying the resistance of LC cells to radiation.

MicroRNAs (miRNA), non-protein coding, single-stranded RNAs of 19–25 nucleotides, constitute a novel class of gene regulators and have been reported to play a critical role in cancer transformation [1]. Recent studies demonstrated the aberrant expression of miRNAs in LC [2]-[5]. The production of miRNAs requires a set of proteins collectively referred to as the miRNA machinery. Aberrant expression of components of the miRNA machinery has been implicated in tumorigenesis, including LC [6], [7]. Up-regulation of Dicer in lung adenocarcinoma and its possible role in the development of peripheral adenocarcinomas have been reported [7]. High expression of other RNA-induced silencing complex (RISC) proteins, fragile X mental retardation syndrome-related protein 1 (FXR1), Tudor-SN (TSN) and protein activator of the interferon-induced protein kinase (PACT), have been demonstrated in SCLC [7]. Another group described an association between reduced Dicer expression and poor prognosis in LC patients [8]. Thus, additional investigations are required to further elucidate the role of miRNA machinery in the molecular pathogenesis of LC. Recently, the potential therapeutic effect of Dicer depletion on the chemosensitivity and proliferation of breast cancer cells has been reported. The knock-down of Dicer by siRNA led to significant G1 arrest and increased sensitivity to the DNA-damaging agent, cisplatin, in the breast cancer cell line MCF-7 [9]. Given the fundamental and multiple biological roles of miRNAs in different cellular processes, the modulation of expression of proteins involved in miRNAs biogenesis might be a promising therapeutic approach for further clinical application. To date there are no data concerning the role of miRNA-producing proteins in mechanisms of resistance/sensitivity of LC cells to treatment. Therefore, we investigated whether the depletion of core proteins involved in miRNAs biogenesis influences the resistance of LC to radiotherapy. Surprisingly, knock-down of expression of Drosha, Dicer, Argonaute2 and Tudor-SN by RNA interference did not increase the sensitivity of NSCLC cells that were resistant to treatment with ionizing radiation.

## Materials and Methods

### Cell Culture and Treatments

Human NSCLC cell lines U1810, U1299 (both from the UU collection), A549, H661, H157, H23 (all from the ATCC); and SCLC cell lines H69 (ECACC), H82 (ATCC), U1906, U1690, U2020, U1285 (all from the UU collection) were maintained in RPMI 1640 medium supplemented with 10% heat-inactivated fetal bovine serum (FBS), glutamine (2 mM), penicillin (100 U/ml) and streptomycin (100 μg/ml) (all obtained from Gibco) at 37°C, 5% CO_2_ and 95% humidity. Cells were exposed to irradiation at a dose of 8 Gy using a ^60^Co source (Karolinska Biomics Center, Karolinska University Hospital) for the periods indicated in the figure legends.

### Evaluation of Apoptosis

Apoptosis was determined by the amount of cells in the sub-G1 phase. The cells were trypsinized and resuspended in phosphate buffered saline (PBS) containing 0.1% FBS. A total of 1×10^6^ cells were used for analysis. Cells were pelleted at 2000 rpm and washed once in PBS. The pellet was resuspended in 250 µL ice-cold PBS and mixed with 2 mL ice-cold 70% ethanol dropwise while vortexing. Samples were kept at 4°C for 24 h and afterwards pelleted at 2000 rpm and washed twice with PBS. After the last wash the cells were resuspended in 360 µL PBS containing 100 µg/mL RNase-A (Fermentas) and incubated at 37°C for 1 h. Forty µL of propidium iodide solution (stock at 0.5 mg/mL) was added to the samples followed by incubation for 30 min at room temperature with gentle swinging and protection from light. Cells were analyzed by flow cytometry (FACScan, Becton Dickinson), and data were evaluated using Cell Quest software.

### Caspase Activity Assay

Cells were washed with ice-cold PBS, resuspended in 25 µL PBS, lysed by freezing in liquid nitrogen, incubated with caspase-3-like substrate and analyzed as described previously [10]. Caspase activity was expressed as the fold of increase relative to appropriate controls.

### siRNA Transfection

siRNAs targeting human Dicer1, Drosha, Argonaute2 and non-targeting siRNA as a negative control were obtained from Thermo Scientific Dharmacon® and stored at a concentration of 20 µM. Twenty-four hours before transfection cells were seeded onto 6-well plates in growth media without antibiotics. siRNAs were diluted in 100 µL RPMI 1640 medium (Gibco) and mixed with 1 µL DharmaFECT®1 siRNA Transfection Reagent (Thermo Scientific Dharmacon®). After 20 min of incubation, the complexes were added to the cells to give a final concentration of siRNAs in the medium of 50 nM. Forty-eight hours after transfection, the medium was replaced and cells were subjected to irradiation.

### Western Blot Detection

Cells were lysed using Complete lysis buffer (Roche) plus protease inhibitor cocktail (PIC, Complete-M, Roche). The protein concentration was determined using the BCA protein assay (Pierce). After mixing with Laemmli’s buffer, samples were subjected to SDS-PAGE and Western blotting. For immunodetection, the following antibodies were used: anti-Dicer, anti-cleaved PARP, anti-cleaved caspase-3, anti-cleaved caspase-9 (all obtained from Cell Signaling Technology), anti-Ago2, anti-Drosha (both from Millipore), anti-XPO5, anti-PRKRA (PACT) (both from Abnova), anti-FXR1 (Santa Cruz Biotechnology), anti-β-actin (Sigma-Aldrich), and anti-GAPDH (Trevigen). Horseradish peroxidase-labeled anti-mouse or anti-rabbit secondary antibodies (Pierce) and an enhanced chemiluminescence kit (Western blot detection reagent, GE Healthcare UK Limited) were used for the detection of recognized proteins. Densitometric analysis for quantification of the relative level of protein expression was performed using ImageJ software (http://rsbweb.nih.gov/ij/).

### Real-Time Quantitative PCR 

RNA was isolated from cells using a PureLink™ RNA Mini Kit (Invitrogen). Reverse-transcribed cDNAs from the samples were used as templates. Argonaute2 (Ago2-left ctaccttcccctggaggtctg and Ago2-right cgacctagcagtcgctctga) and 18S ribosomal RNA (18S-1 cgctactaccgattggatggtt and 18S-2 agtcaagttcgaccgtcttctc) primers (Invitrogen) were designed to match the target cDNA sequence. Twenty ng of the reverse-transcribed cDNA template was mixed with SYBR® Green PCR Master Mix (Applied Biosystems) and amplified using a 7500 Real-Time PCR System (Applied Biosystems) with the following program: 40 cycles, with each cycle consisting of a denaturation step at 95°C for 15 s and an annealing/extension step at 60°C for 1 min.

### Statistical Evaluation

The results of three independent experiments were expressed as the mean ± S.E.M. Statistical evaluation was performed using a non-paired t-test.

## Results

### Expression of Proteins Involved in miRNA Biogenesis in NSCLC and SCLC 

**Figure 1:**
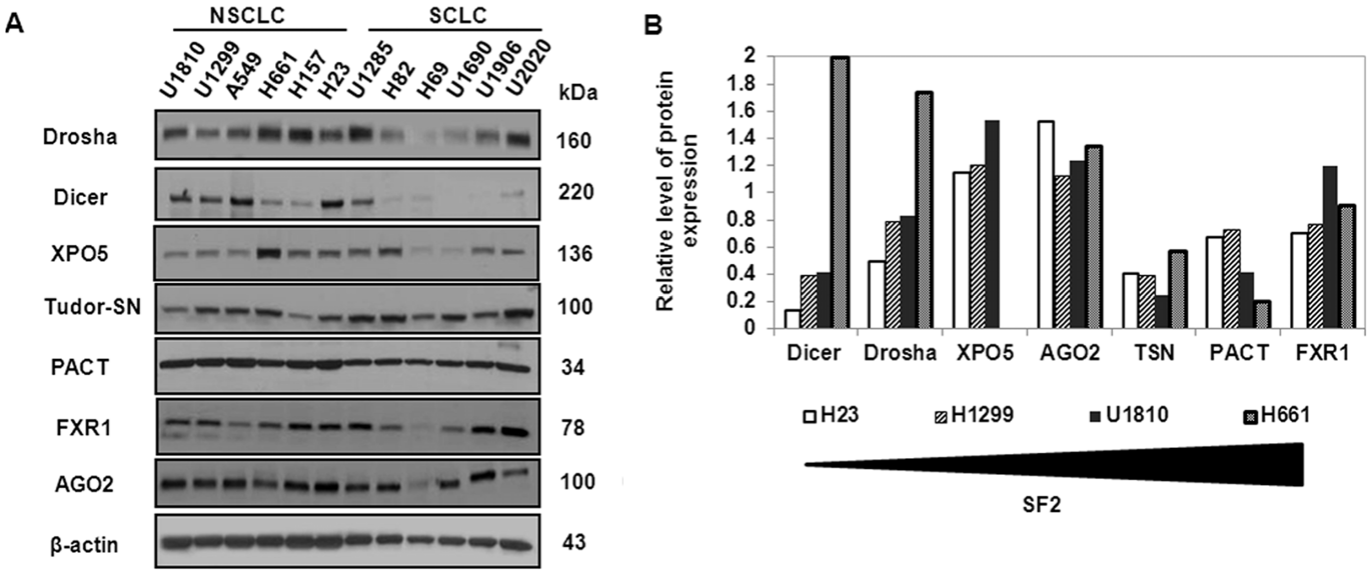
NSCLC and SCLC cells differ in sensitivity to radiation treatment and display differential expression of proteins involved in the regulation of miRNA biogenesis. (A) Western blot analysis of the level of protein expression of Drosha, Dicer, exportin 5 (XPO5), Tudor-SN (TSN), protein activator of the interferon-induced protein kinase (PACT), fragile X mental retardation syndrome-related protein 1 (FXR1) and Argonaute 2 (AGO2) in a panel of NSCLC (U1810, U1299, A549, H661, H157, H23) and SCLC (U1285, H82, H69, U1690, U1906, U2020) cell lines. (B) Densitometric analysis of relative levels of protein expression in H23, H1299, U1810 and H661 cell lines. Cell lines distributed according to radiosensitivity, measured as the fraction surviving at 2 Gy (SF2). Equal loading was verified using anti-β-actin antibodies. Results are representative of three independent experiments.

In order to identify molecular targets for the radiosensitization of LC cells among proteins involved in miRNA biogenesis we performed a protein expression analysis of seven core miRNA machinery components in a panel of NSCLC and SCLC (six cell lines in each panel). For each LC subtype the cell lines were selected based on their radiosensitivity, as measured by the fraction surviving after exposure to 2 Gy (SF2) in a clonogenic survival assay, and grouped into radiosensitive (RS) with SF2 < 0.3 Gy or radioresistant (RR) with SF2 ≥ 0.3 Gy [11]-[14]. The basal levels of all proteins (Drosha, Dicer, exportin-5 (XPO5), Tudor-SN (TSN), protein activator of the interferon-induced protein kinase (PACT), fragile X mental retardation syndrome-related protein 1 (FXR1) and Argonaute2 (Ago2) were assessed by Western blot in all selected cell lines prior to irradiation (Figure 1A). Both RNase III enzymes (Drosha and Dicer) were expressed at relatively high levels in NSCLC cells compared with SCLC. A member of the karyopherins protein family, XPO5, which is involved in the nuclear export of miRNAs, was expressed at a higher level in H661 while low levels of expression were seen in H69 and U1690 compared to the remaining cell lines. The level of expression of TSN, PACT, FXR1 and Ago2 proteins did not vary profoundly among the various cell lines, with the exception of H69, which exhibited lower expression of all of the studied proteins. As our panel consisted of both RS and RR cells, the H23 cell line was selected as the representative of radiosensitive cells, and U1810 and H661 were used as representatives of radioresistant cells in further investigations. Densitometric analysis of protein expression revealed that Dicer, Drosha and XPO5 were expressed at higher levels in RR cells compared to RS, whereas there was no clear correlation between the levels of expression of Ago2, TSN, PACT and FXR1 and SF2 values (Figure 1B).

### Effect of Ionizing Radiation on miRNA Machinery in NSCLC 

**Figure 2:**
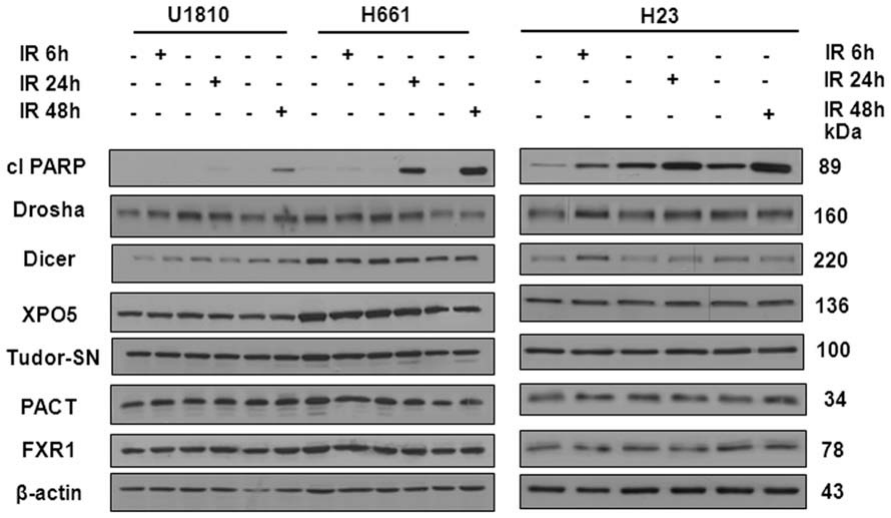
Expression of components of miRNA machinery in NSCLC following treatment with ionizing radiation. The cleavage of PARP and the level of expression of Drosha, Dicer, exportin 5 (XPO5), Tudor-SN (TSN), protein activator of the interferon-induced protein kinase (PACT), and fragile X mental retardation syndrome-related protein 1 (FXR1) in U1810, H661 and H23 cells were detected by Western blot at 6, 24 and 48 h post-irradiation with 8 Gy. Equal loading was verified using anti-β-actin antibodies. Data are representative of three independent experiments.

To further investigate the role of miRNA machinery components in the response of LC cells to irradiation, two RR cell lines (U1810 and H661) and one RS line (H23) from the panel of NSCLC were subjected to gamma irradiation and protein expression was analyzed 6, 24 and 48 h after treatment. As expected, massive apoptotic cell death was observed in H23 at 6 h after ionizing radiation, as assessed by the cleavage of PARP, whereas H661 and U1810 responded to treatment at later time points after 24 and 48 h, respectively (Figure 2). No visible changes in expression of any of the studied proteins were detected either in RR or RS cells, as measured by Western blot at 6, 24 and 48 h after IR treatment (Figure 2). Thus, gamma irradiation does not affect the expression of core proteins of the miRNA machinery in NSCLC regardless of the RR or RS phenotype of these cancer cells.

### Knock-down of Core Proteins Involved in the First Step of miRNA Biogenesis is not Sufficient to Sensitize NSCLC Cells to Irradiation

Since the level of protein expression of at least three miRNA machinery components, Dicer, Drosha and XPO5, was positively correlated with the radioresistance of NSCLC cells, the high level of their expression may contribute to the tumor cells’ response to treatment in a miRNA-guided fashion. To explore this possibility we decided to investigate the potential sensitizing effect of down-regulation of Dicer and Drosha proteins in the U1810 cell line. The nuclear ribonuclease Drosha and the cytoplasmic ribonuclease Dicer are the two main regulators of the first step of miRNA production and promote the cleavage of long primary transcripts into the hairpin intermediates (pre-miRNAs) and subsequent cleavage into mature miRNAs. Thus, by eliminating either of these two core proteins we expected to reduce miRNA production and affect the radioresistance of NSCLC cells.

**Figure 3:**
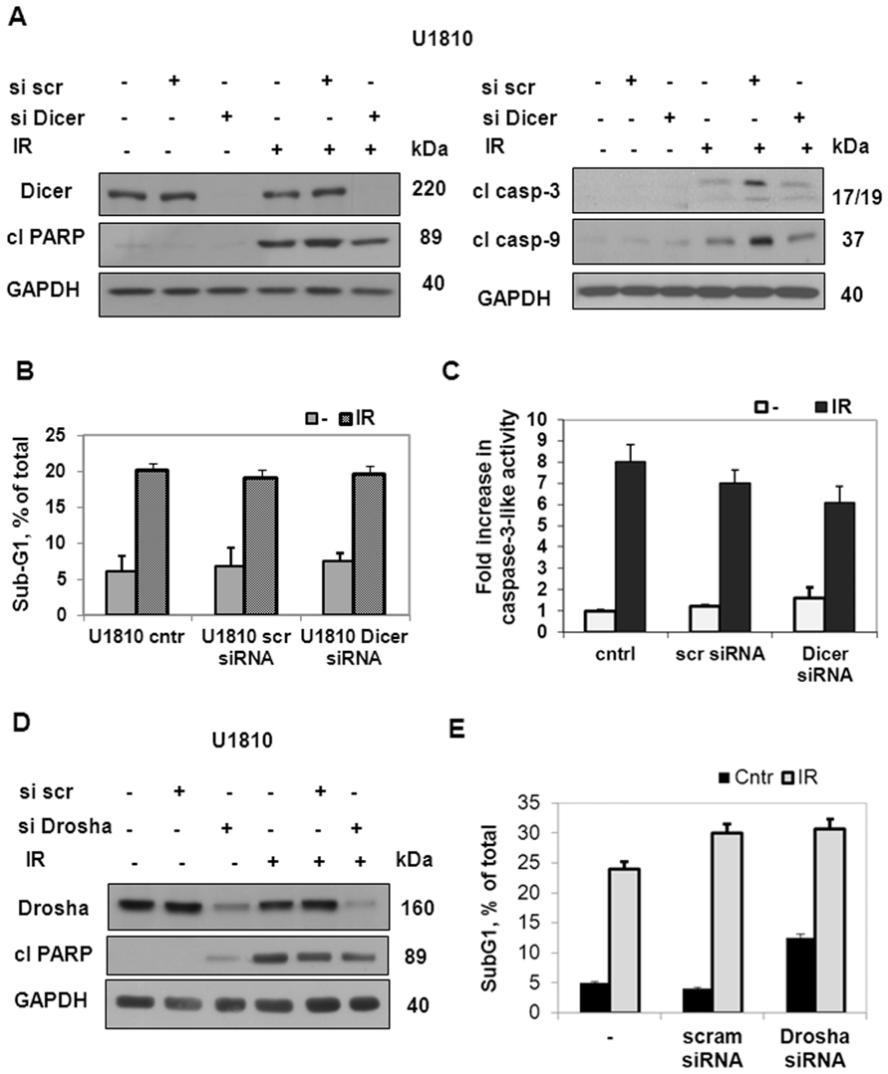
Knock-down of Dicer and Drosha is not sufficient to sensitize NSCLC cells to irradiation. (A) The level of Dicer expression, cleavage of PARP and processing of caspase-3 and -9 in U1810 cells transfected (48 h) with control (si scr) or Dicer (siDicer) siRNA assessed by Western blot 48 h after irradiation. Equal loading was verified using anti-GAPDH antibodies. Data are representative of three independent experiments. (B) Detection of apoptotic cell death in U1810 assessed by measuring the sub-G1 population after transfection (48 h) with control or Dicer siRNA and irradiation treatment (48 h). (C) Caspase-3-like activity (fold increase with respect to control) in U1810 cells after treatment with either irradiation alone or in combination with transfection of control or Dicer siRNA (for details see Materials and methods). (D) The level of Drosha expression and cleavage of PARP in U1810 cells transfected (48 h) with control (si scr) or Drosha (siDrosha) siRNA analyzed by Western blot 48 h after treatment with irradiation. Equal loading was verified using anti-GAPDH antibodies. All data are representative of three independent experiments. (B) Apoptotic cell death in U1810 measured by analysis of the sub-G1 population after transfection (48 h) with control or Drosha siRNA and irradiation treatment (48 h). Results shown are the mean±S.E.M. of three independent experiments.

The knock-down of Dicer in U1810 cells was performed using RNA interference, after which the cells were subjected to gamma irradiation and analyzed 48 h after treatment. The complete silencing of Dicer protein expression was confirmed by Western blot before and after irradiation (Figure 3A). Down-regulation of Dicer was followed by decreased production of several miRNAs (miR1301, miR1249, miR1227, miR532-3p, miR625, miR1827, miR324-5p) as assessed by real-time PCR (data not shown). However, U1810 cells with depleted Dicer exhibited as strong response to ionizing radiation as did wild-type cells. There were no differences in the cleavage of PARP between control and Dicer knocked-down cells (Figure 3A). The percentage of apoptotic cells after irradiation was the same in all studied groups (Figure 3B). Analysis of activation of caspases showed that caspase-3 and -9 were equally processed after irradiation and that caspase-3-like activity was almost identical in both wild-type and Dicer-depleted cells (Figure 3A and C). A similar effect on radioresistance was apparent when Drosha was depleted in U1810 cells. No changes in PAPR cleavage (Figure 3D) or the number of apoptotic cells (Figure 3E) were detected among control and Drosha knocked-down cells 48 h after ionizing radiaiton. Similar results were obtained following the elimination of Dicer and Drosha from other NSCLC cell lines (Figure S1 and S2). Overall these data suggest that knock-down of either Dicer or Drosha was not sufficient to sensitize NSCLC cells to gamma irradiation.

### Down-regulation of Main Components of RNA-induced Silencing Complex (RISC) does not Sensitize NSCLC Cells to Irradiation

**Figure 4:**
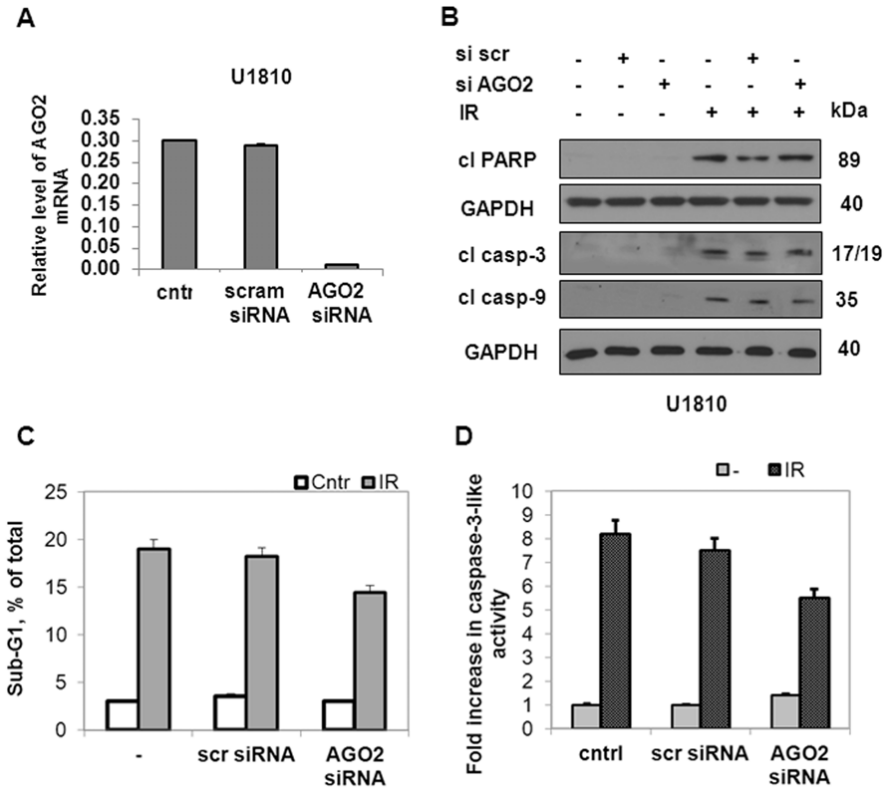
Depletion of a component of the RNA-induced silencing complex (RISC), Argonaute2, does not affect the radioresistance of NSCLC. (A) The level of Argonaute2 (AGO2) mRNA in U1810 cells transfected with control (scram) or AGO2 siRNA normalized against 18S ribosomal RNA. Results are the mean±S.E.M. of three independent experiments. (B) Cleavage of PARP and processing of caspase-3 and -9 in U1810 cells transfected (48 h) with control (si scr) or AGO2 siRNA, and then subjected to irradiation for 48 h. Equal loading was verified using anti-GAPDH antibodies. All data are representative of three independent experiments. (C) Detection of apoptotic cell death in U1810 cells after transfection (48 h) with control or AGO2 siRNA and subsequent treatment with irradiation (48 h). (D) Caspase-3-like activity (fold increase with respect to control) in U1810 cells after treatment with either irradiation alone or in combination with transfection with control or AGO2 siRNA. All results shown are the mean±S.E.M. of three independent experiments.

Since the depletion of core proteins of the first step of miRNA biogenesis did not affect the radiosensitivity of NSCLC, it is possible that RNA interference-mediated knock-down of either Dicer or Drosha results in a significant reduction, but not the full loss, of mature miRNA due to the long half-life of mature molecules. miRNAs are stable once they enter into the effector complex. Therefore, we decided to block the second step of miRNA biogenesis by knock-down of the two main components of RISC, Argonaute2 and Tudor-SN, and thus establish their role in the radioresistance of LC cells. The effective down-regulation of expression of Ago2 in the U1810 cell line was confirmed by measuring its mRNA expression, which was reduced by up to 95% after transfection with anti-Ago2 siRNA (Figure 4A). Nevertheless, the apoptotic response (assessed by PARP cleavage and processing of caspase-3 and -9) exhibited by Ago2-depleted cells after irradiation was as strong as in the wild-type U1810 cells (Figure 4B). Additionally, there were no significant changes in the percentage of the sub-G1 population or the activation of caspase-3 after ionizing radiation, although both parameters were slightly decreased in Ago2-down-regulated cells compared to wild-type cells (Figure 4C and D).

**Figure 5:**
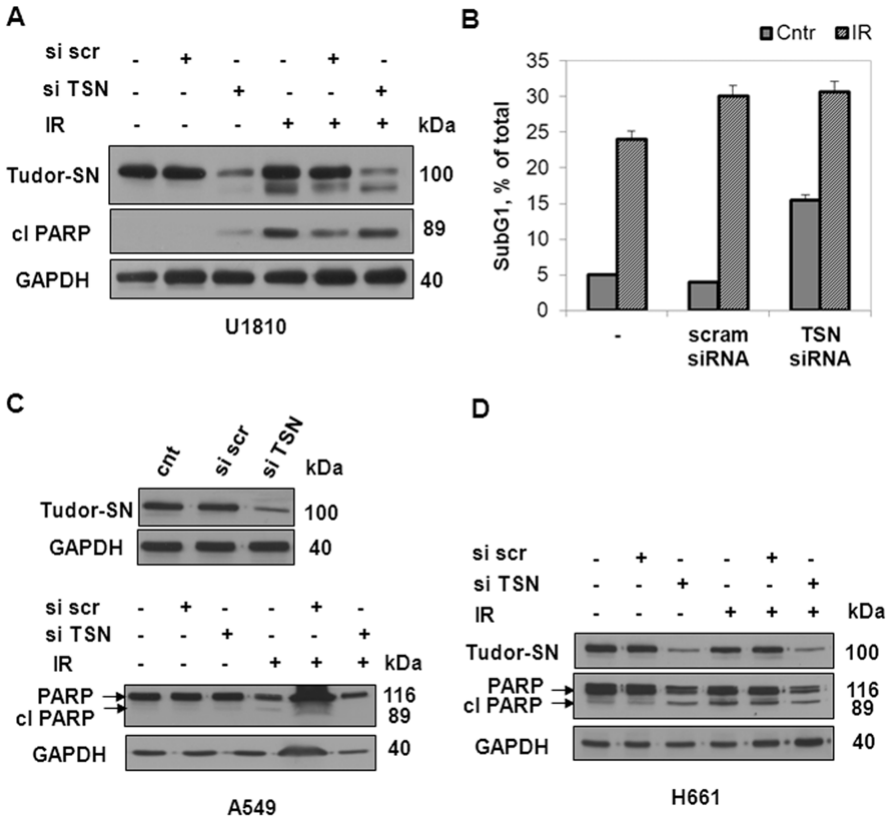
The knock-down of a component of the RNA-induced silencing complex (RISC), Tudor-SN, is insufficient to sensitize NSCLC to irradiation. The level of Tudor-SN expression and cleavage of PARP in U1810 (A), A549 (C) and H661 (D) cells transfected (48 h) with control (si scr) or Tudor-SN (si TSN) siRNA analyzed by Western blot 48 h after irradiation. Equal loading was verified using anti-GAPDH antibodies. (B) The apoptotic cell death in U1810 cells after transfection with TSN siRNA and irradiation. All data are representative of three independent experiments.

Finally, the same set of experiments was conducted in order to knock-down the expression of another RISC component, Tudor-SN. Besides its function as a component of the multiprotein complex involved in miRNA functioning, TSN is known to act as a transcriptional activator and oncogene in many cancers. Additionally, it is cleaved during apoptosis. Even though significant down-regulation of TSN expression using siRNA was achieved in U1810 cells, as shown by Western blot analysis, the knocked-down cells exhibited similar cleavage of PARP as the wild-type upon ionizing radiation treatment (Figure 5A). The percentage of apoptotic cells did not differ between control and TSN knocked-down groups after irradiation (Figure 5B). Similar results were obtained for two other cell lines from the NSCLC panel (A549 and H661) after down-regulation of TSN (Figure 5C and D).

## Discussion

Radiotherapy is a major therapeutic weapon in LC. However, the effectiveness of this type of therapy is limited by the initial or acquired radioresistance of the tumor cells. NSCLC is characterized by a low tumor response rate to irradiation and a 5-year survival rate of only 7% to 10% [15]. SCLCs initially respond well to conventional chemo- and radiotherapy, but develop acquired chemo- and radioresistance over the subsequent 3 to 12 months, and the overall 5-year survival rate is only 5% [16]. The mechanisms leading to the radioresistance of these tumors are not yet fully understood.

miRNAs have been reported to be potential diagnostic or therapeutic targets in cancer treatment, including lung tumors. There is increasing evidence of the association between miRNA expression in tumors and chemo- and radiosensitivity, both with regards to predicting and modulating sensitivity [17]-[20]. A recent study on NSCLC identified a subset of miRNAs showing robust changes in expression in response to irradiation. Thus, a global miRNA response does exist in all tumor cells, including lung cancer, and miRNAs might be components of the cellular response to cytotoxic insult [20]. Similar findings concerning the changes in global miRNA expression were reported after anticancer treatment with various chemotherapeutic drugs in different cancer cell lines and patient samples [21]. The expression of the miRNA processing components was shown to be deregulated in different human cancers [8], [22], [23] and, thus, might contribute to tumor cells’ response to treatment in a miRNA-guided fashion or independently of the RNA interference pathway. Down-regulation of Dicer in MCF-7 breast cancer cell line by siRNA was shown to cause G1 arrest and increase sensitivity to the DNA-damaging agent, cisplatin, suggesting that the combination of the anti-Dicer strategy and traditional chemotherapy could improve the efficiency of cancer treatment [9]. Another study demonstrated that Dicer down-regulation can increase the proliferative and invasive ability of tumor cells in vitro and similarly promote subcutaneous tumor xenograft proliferation in vivo 24. Overall, these data suggest that miRNA machinery plays either a negative or positive role in tumor transformation and response to therapy in different types of cancer. In the present study, we aimed to clarify the role of miRNA biogenesis components in the modulation of radiation response in NSCLC and SCLC cell lines. In order to identify molecular targets for radiosensitization, the protein expression analysis of seven core proteins of the miRNA machinery, Drosha, Dicer, XPO5, TSN, PACT, FXR1 and Ago2, was performed in a panel of NSCLC and SCLC cell lines. Our findings demonstrated the higher expression of Drosha and Dicer proteins in cells resistant to radiation compared to sensitive lines. However, knock-down of these two essential proteins did not affect the sensitivity of NSCLC cells to treatment with gamma irradiation. This may partially be due to the long half-life of mature miRNA. Several studies have shown that RNA interference-mediated knock-down of Drosha, XPO5-5 or Dicer results in a significant reduction, but not the full loss, of mature miRNA [25]-[28]. Since miRNAs seem to be quite stable once they enter into the effector complex, we explored the possibility of increasing the radiosensitivity of lung tumor cells by the depletion of down-stream components of the miRNA biogenesis pathway, i.e., two main RISC proteins, Ago2 and Tudor-SN. Nevertheless, down-regulation of these proteins did not affect the sensitivity of NSCLC cells to treatment. So, there may be alternative biogenesis pathway(s) that can be activated upon disruption of one of the multiple steps in the canonical miRNA biogenesis process. Some miRNAs were found to be generated via a Dicer-independent biogenesis pathway: after being processed by Drosha, the pre-miRNAs can be directly loaded to Ago and cleaved by the Ago catalytic center to generate an intermediate 3̀ end, which is further trimmed [29]. There is also a class of non-canonical miRNAs that bypass the Drosha microprocessor, but still require Dicer for their biogenesis [30]. It should be noted that analysis of the protein expression of all core components of the miRNA machinery in U1810 cells performed after down-regulation of Dicer, Drosha, TSN or Ago2 revealed that neither of the knock-downs had a significant effect on the expression of other proteins in the pathway, i.e. we observed no increase in the expression of miRNA machinery components following knock-downs (Figure S3). On the other hand, a recent study performed on immortalized and primary endothelial cells showed that global suppression of miRNA expression achieved through down-regulation of either Ago2 or Dicer proteins using siRNA led to increased cell death after irradiation [31]. This indicates that the contribution of the miRNA machinery to the response to radiotherapy might highly depend on cell type and be different in normal and cancer cells.

Another report showed that miRNA biogenesis is globally induced upon DNA damage in an ataxia-telangiectasia mutated (ATM) kinase-dependent manner in mouse embryonic fibroblasts (MEFs) after treatment with the radiomimetic drug neocarcinostatin (NCS), which generates double-strand breaks (DSBs). KH-type splicing regulatory protein (KSRP) was found to be a key player that translates DNA damage signaling to miRNA biogenesis. The ATM kinase directly binds to and phosphorylates KSRP, leading to enhanced interaction between KSRP and pri-miRNAs and increased KSRP activity in miRNA processing [32]. Whether KSRP is activated and contributes to miRNA processing in NSCLC upon the down-regulation of core components of the miRNA machinery and treatment with gamma irradiation remains to be clarified.

Additionally, it is known that most miRNAs have dozens to hundreds of targets, and that target mRNAs may bind multiple miRNAs. Perhaps the magnitude of decrease in miRNA production and activity caused by the elimination of a single protein from a miRNA pathway is not enough to affect the mechanisms responsible for the resistance of LC cells to irradiation treatment. In other words, it is likely that a real impact on the radioresistance of NSCLC cells via the miRNA machinery cannot be achieved by the elimination of single proteins from the biogenesis pathway, and thus further identification and targeting of particular miRNAs involved in the response of LC cells to irradiation therapy is required.

In summary, in the present study the expression of a set of proteins involved in miRNA biogenesis was assessed for the first time in a panel of human LC cell lines. Even though the expression of core proteins of the miRNA pathway correlated with the resistance of cells to radiotherapy, the knock-down of these proteins was not sufficient to trigger the sensitization of LC cells to this type of treatment. This suggests that lung tumor radioresistance cannot be overcome by modulation of the miRNA biogenesis machinery and that other strategies are required to combat lung cancer.

## Supporting Information

Figure S1 The expression of Dicer and PARP cleavage in A549 and H661 cells transfected with control (si scr) or Dicer (si Dicer) siRNA analyzed by Western blot 48 h after treatment with irradiation (A). Equal loading was verified using anti-GAPDH antibodies. (B) Apoptotic cell death in A549 and H661 cells measured by analysis of the sub-G1 population after transfection (48 h) with control or Drosha siRNA and irradiation treatment (48 h).
(TIF)

Figure S2 The level of Drosha and cleaved PARP in A549 (A) and H661 (C) cells after knock-down of Drosha. Equal loading was verified using anti-GAPDH antibodies. (B) The percentage of apoptotic cells in A549 transfected with Drosha siRNA and treated with irradiation (48 h). (D) Caspase-3-like activity (fold increase with respect to control) in H661 cells after treatment with either irradiation alone or in combination with transfection with control or Drosha siRNA.
(TIF)

Figure S3 The level of Drosha, Dicer, XPO5, TSN, PACT after knock-down of Dicer, Drosha, TSN and Ago2 in U1810 cells. Equal loading was verified using anti-GAPDH antibodies.
(TIF)
