# Supplementary figures and images for: Knock-Down of Core Proteins Regulating MicroRNA Biogenesis Has No Effect on Sensitivity of Lung Cancer Cells to Ionizing Radiation
Source: PLoS One. 2012 Mar 30;7(3):e33134. doi: 10.1371/journal.pone.0033134 (PMC3316564; doi:10.1371/journal.pone.0033134)

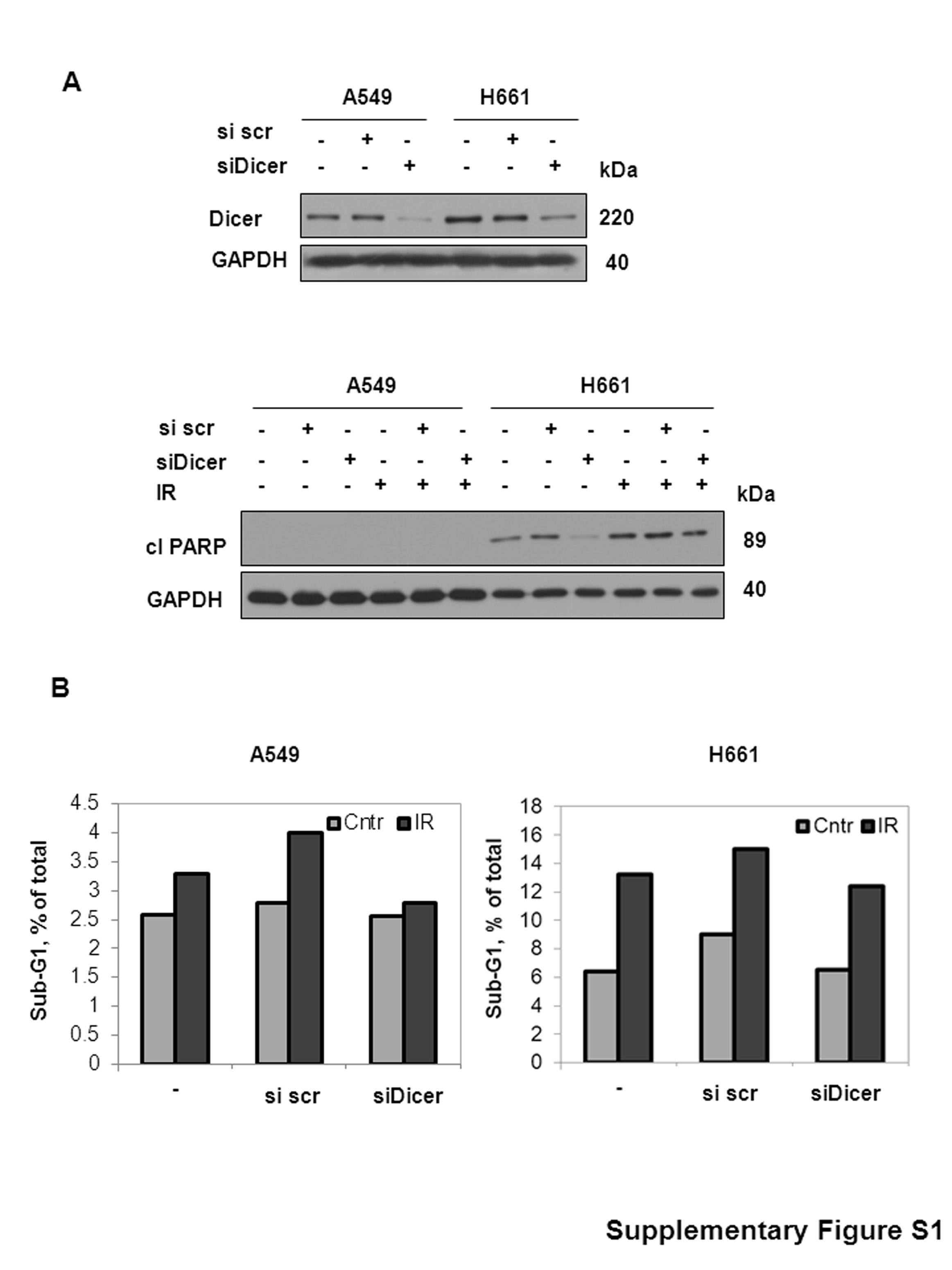

Supplement: Figure S1 — The expression of Dicer and PARP cleavage in A549 and H661 cells transfected with control (si scr) or Dicer (si Dicer) siRNA analyzed by Western blot 48 h after treatment with irradiation (A). Equal loading was verified using anti-GAPDH antibodies. (B) Apoptotic cell death in A549 and H661 cells measured by analysis of the sub-G1 population after transfection (48 h) with control or Drosha siRNA and irradiation treatment (48 h). (TIF) [file pone.0033134.s001.tif]

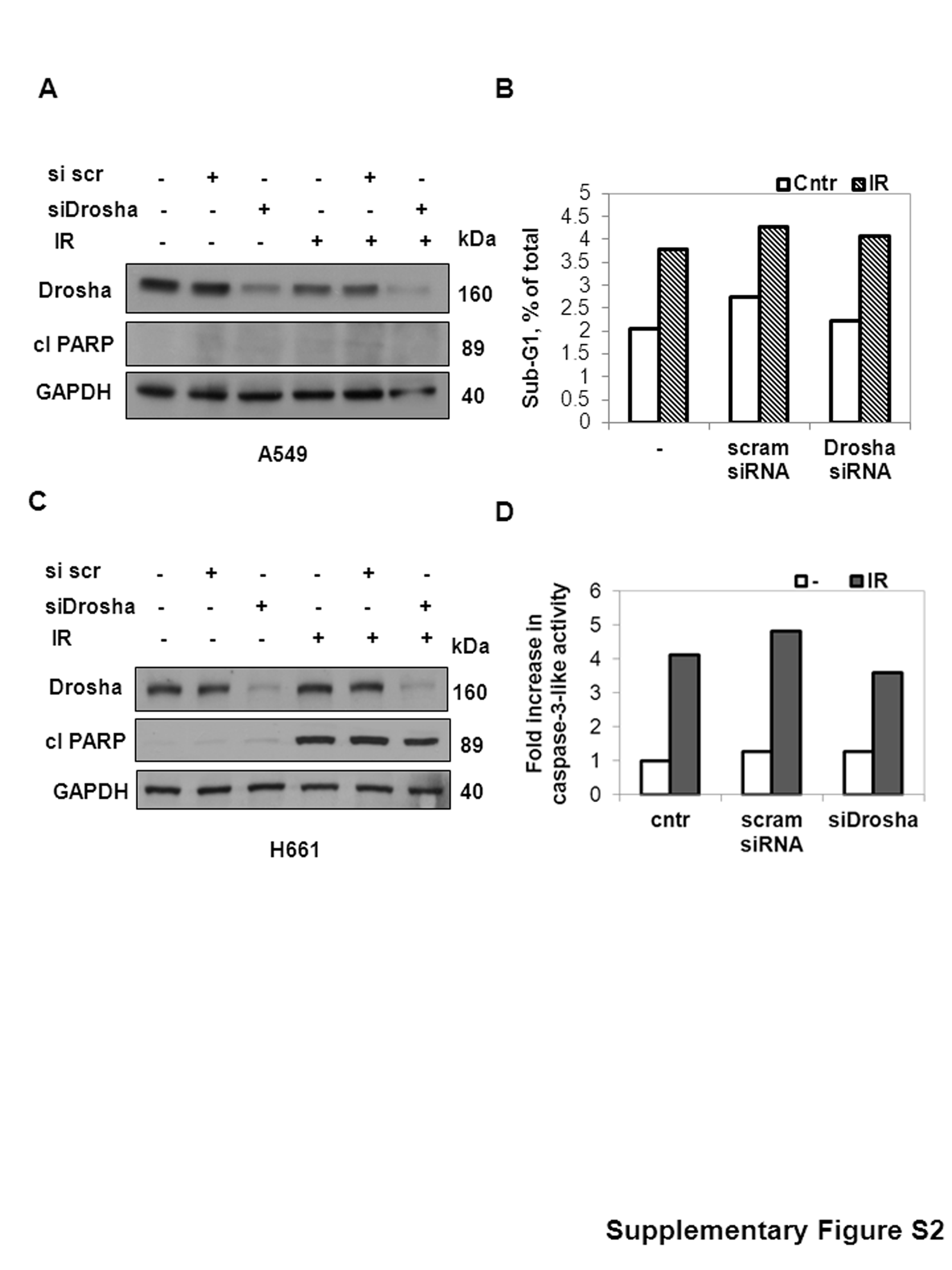

Supplement: Figure S2 — The level of Drosha and cleaved PARP in A549 (A) and H661 (C) cells after knock-down of Drosha. Equal loading was verified using anti-GAPDH antibodies. (B) The percentage of apoptotic cells in A549 transfected with Drosha siRNA and treated with irradiation (48 h). (D) Caspase-3-like activity (fold increase with respect to control) in H661 cells after treatment with either irradiation alone or in combination with transfection with control or Drosha siRNA. (TIF) [file pone.0033134.s002.tif]

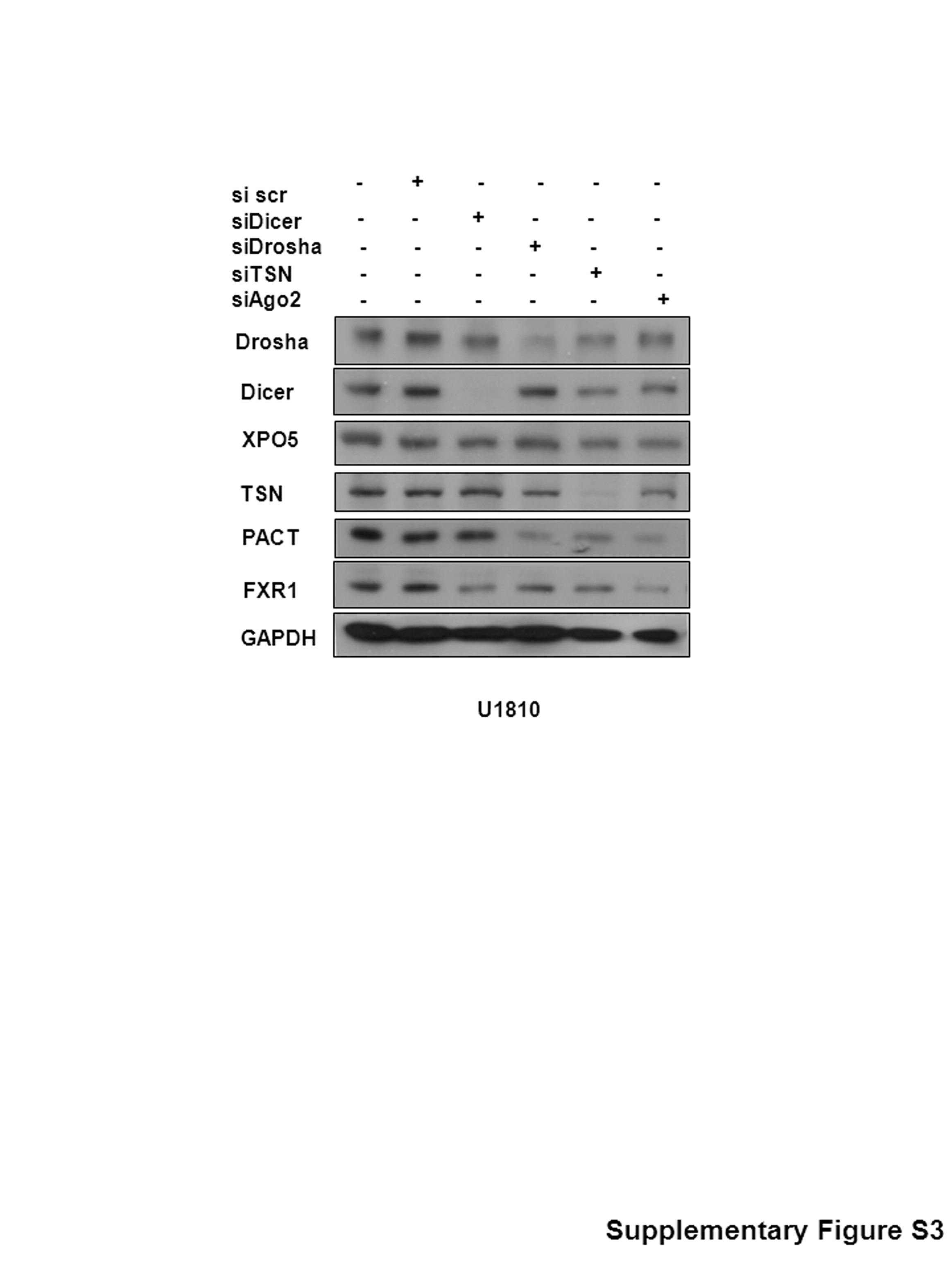

Supplement: Figure S3 — The level of Drosha, Dicer, XPO5, TSN, PACT after knock-down of Dicer, Drosha, TSN and Ago2 in U1810 cells. Equal loading was verified using anti-GAPDH antibodies. (TIF) [file pone.0033134.s003.tif]
